# eSRT Versus Population Mean: Setting Upper Stimulation Levels in Adult Cochlear Implant Users

**DOI:** 10.1097/ONO.0000000000000071

**Published:** 2025-05-28

**Authors:** Jourdan T. Holder, Andrina MacDonald, René H. Gifford

**Affiliations:** 1 Department of Hearing and Speech Sciences, Vanderbilt University Medical Center, Nashville, Tennessee; 2 Hearts for Hearing, Oklahoma City, Oklahoma.

Cochlear implants (CIs), when programmed correctly, provide sufficient detail to allow for a full range of acoustic percepts and support speech understanding in quiet and noise for most recipients. Upper stimulation levels (USLs) are arguably the most important modifiable parameter for a CI user’s programming as they are responsible for the transmission of mid- to high-level sounds. If USLs are set too low, this can negatively impact loudness discrimination, speech recognition in noise, sound quality, and ability to monitor one’s own voice (1–5). If USLs are set too high, this can result in loudness discomfort for typical sound levels and can negatively impact sound quality and speech recognition (6,7).

Seventy-one (71%) percent of clinicians use a psychophysical loudness scaling task to set USLs where the patient rates the loudness of a beeping stimulus played through the implant (8). This method is problematic as many patients are unable to accurately complete this task due to factors such as duration of deafness, age, and cognitive status (2,9–11). Before pursuing implantation, the typical adult CI recipient has had hearing loss for 27 years (12) with 8–10 years of severe-to-profound deafness (12,13). Despite long durations of hearing loss, we ask patients to characterize the loudness of stimuli they may not have heard in decades. Consequently, it is common for adult recipients with acquired hearing losses to set USLs low, particularly for basal electrodes conveying high-frequency information (14,15).

The electrically evoked stapedial reflex threshold (eSRT) is the gold standard for setting USLs, as it is an objective measure that gives clinicians a target for USLs. eSRTs are useful for setting USLs because the reflex is an objective measurement elicited in response to a stimulus that is perceived as loud, yet not uncomfortable (14). The strong correlation (r = 0.80–0.86) between eSRTs and USLs has been recently described in detail (16,17) with the average difference between USLs and eSRT being specific to each CI manufacturer based on programming differences (15.4 clinical levels below eSRT for Cochlear). Further, studies have shown eSRTs and associated USLs to be stable from 1- to 6-month postactivation (16,17). Indeed, eSRT-based USLs are consistently higher than that achieved via behavioral loudness scaling (18,19,20), resulting in a broader electrical dynamic range (EDR). A broader EDR is at least partially responsible for the superiority of eSRT-based maps, which can result in significantly higher speech understanding (14,21) and subjective sound quality (14). However, there are other likely variables at play including a flatter USL profile with higher USLs, particularly for basal electrodes (22).

One disadvantage to eSRTs is that they are not always able to be obtained due to equipment, time, audiologist familiarity, patient compliance/tolerance, and middle ear status. In light of this disadvantage, we investigated the use of population mean mapping, a Cochlear (NSW, Australia) tool readily available in the software to guide the setting of USLs without any additional steps such as psychophysical loudness scaling or use of an objective measure, like eSRT. Population mean is thought to provide a valuable reference point based on average USLs for ~15,000 maps from multiple centers (23). However, because it is well-known that clinicians use a variety of different techniques to fit USLs—including electrically evoked compound action potentials, loudness scaling, and eSRTs—much of the clinical data informing the population mean calculation may result in the underfitting of USLs, particularly for basal electrodes (14,15,24,25). This study investigated the relationship between USLs obtained via population mean and eSRTs, the latter being an evidence-based method for setting USLs. The primary aim of this study was to evaluate the relationship between eSRT and Cochlear’s population mean that is currently available in the programming software. Based on previous reports of clinicians tending to underfit patients (14,15,24,25), we hypothesized that USLs based on eSRT would be higher than USLs based on population mean.

## METHODS

Methods were approved by our institutional review board (IRB 180939). A retrospective review was conducted for patients who had eSRTs measured during their 1-month postactivation clinical appointment; measurement of eSRTs at 1-month postactivation is standard of care at our center. eSRTs and resulting USLs for maps created at the 1-month and 6-month postactivation time point were collected for 5 electrodes across the array (2, 6, 11, 16, 22). Electrode 2 (instead of electrode 1) was chosen due to lower likelihood of being extracochlear and/or deactivated, and thus more available datapoints for electrode 2. Values for all participants on all 5 electrodes were not always available for various reasons such as extracochlear electrodes, absent eSRT, deactivated electrodes, etc. Additionally, fewer datapoints were available at 6 months compared to 1 month because patients did not return for a 6-month follow-up visit as consistently. Participants were postlingually deafened adult (mean age = 61 years) recipients. Methods for this data collection are explained in detail in Holder et al. The purpose of this brief communication was to compare already published data to the population mean USLs in the Cochlear Custom Sound software. Population mean USLs and associated standard deviations were derived from Maruthurkkara et al, as well as personal communication with Saji Maruthurkkara and the Cochlear team. To align our data as closely as possible to the population mean data, only maps using a default rate of 900 Hz and a default pulse width 25 μs for 532/632 and 37 μs for 522/622 were included in this brief report, as rate and pulse width are known to affect eSRTs and USLs (20). Population mean data differ by electrode type due to the known relationship between electrode to modiolus distance and charge required for USLs (23).

## RESULTS

**FIG. 1.**
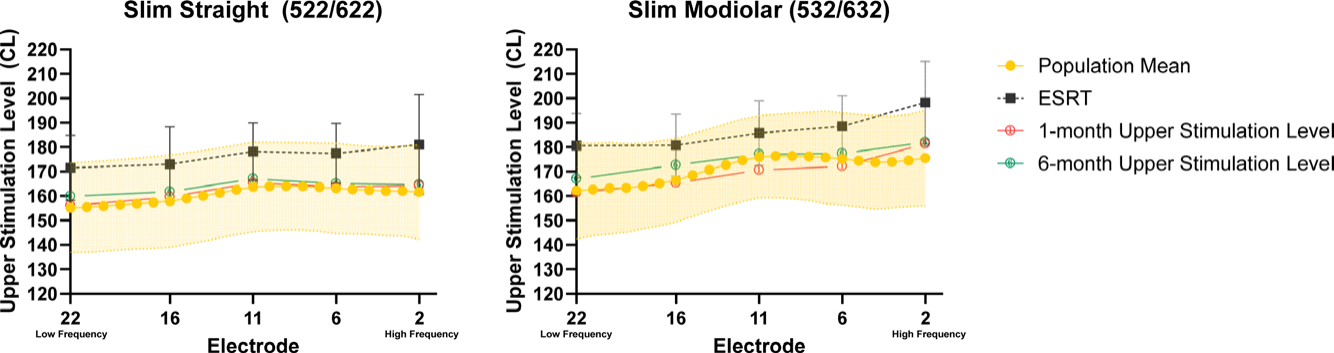
Electrically evoked stapedial reflex thresholds (eSRTs) measured at 1-month postactivation, as well as, upper stimulation levels at the 1- and 6-month postactivation appointments are shown. The error bars represent standard deviation. Population mean and ±1 standard deviation are indicated by the shaded area.

Data were available for 177 Cochlear recipients. Of those, 101 had Slim Straight/522/622 devices and 76 had Slim Modiolar/532/632 devices. Contour Advance/512/612 electrodes were not included due to a much lower available sample (n = 26). Figure 1 and Table 1 display mean eSRTs measured at 1-month, as well as the resulting USLs used in the patient’s map at 1- and 6-month postactivation with the population mean ± 1 standard deviation indicated by the shaded area on the graph. The shape of the eSRT-level profile, resultant USLs, and population mean all have a similar rising profile. Specifically, 612/792 (77.3%) USLs at 1 month and 386/492 (78.4%) USLs at 6 months fell within ± 1 standard deviation of the population mean.

**TABLE 1.** Population mean values, population mean standard deviation values, electrically evoked stapedial reflex thresholds (eSRTs), as well as, upper stimulation levels (USLs), at the 1- and 6-month postactivation appointments are shown for each electrode for the 2 electrode array types

	Slim straight (522/622)				
Electrode	22	16	11	6	2
Population Mean	155	158	164	163	162
Population Mean Std Dev	18	19	18	19	19
eSRT	172	173	178	177	181
1-month USL	156	160	165	164	164
6-month USL	160	162	167	165	165
	Slim modiolar (532/632)				
Electrode	22	16	11	6	2
Population Mean	162	166	176	175	175
Population Mean Std Dev	20	19	17	18	19
eSRT	181	181	186	189	198
1-month USL	161	165	171	172	181
6-month USL	167	173	177	177	182

All values are clinical levels (CLs).

## DISCUSSION

Prior studies have shown eSRTs to be the superior method of programming USLs for adults and children (3,14,26,18), allowing for individual programming optimization without reliance upon often biased or erroneous patient report. Despite converging evidence that eSRTs are the gold standard for this programming parameter, many clinicians do not have appropriate training, equipment, or time to implement this measure. Further, we know that eSRTs cannot be measured on all patients for a variety of reasons (27,28). Thus, the purpose of this study was to examine the relationship between eSRTs, USLs, and Cochlear’s population mean to assess the efficacy of using population mean to guide USL programming.

The data shown in Figure 1 suggest that USLs set based on eSRTs obtained at 1-month and 6-month postactivation are in good agreement with the population mean range of clinical levels and USL shape or profile. The fact that 77% of USLs fell within ±1 standard deviation of the population mean suggests that using population mean to guide USLs is a method that can offer similar USLs to those obtained via evidence-based eSRTs, which is particularly useful when eSRTs are not available due to equipment, time, or patient limitations. This finding is favorable for programming audiologists because population mean is readily available and does not require additional assessment; however, eSRTs continue to be the preferred programming method as it fully accounts for individual variability and is the only evidence-based method for USL programming demonstrating significantly higher patient outcomes (14,21).

The authors recommend implementing these findings in a complementary clinical workflow. The clinician can use the population mean to create the patient’s initial stimulation map, then increase T and C levels globally in live voice mode until the patient reports that voices are “too loud.” After a few minutes of acclimatization, if voices are still too loud, reduce C levels slightly. This practice ensures that USLs are not set too low and the EDR is optimized. If needed, the recipient could be given a series of progressively louder maps where the last and loudest map approximates the population mean USLs, or the recipient could be given access to the master volume control. At the first follow-up visit, we recommend measuring eSRTs to further optimize and individualize the patient’s programming. Given the results herein, minimal adjustments should be expected based on eSRT to achieve optimization at the first follow-up visit, which is ideal for our overall goal of optimizing a patient’s access to the full dynamic range as soon as possible. If eSRTs cannot be obtained, the data presented herein support the use of the population mean USLs shaded region on the mapping function screen as a guideline for USLs combined with patient report.

Cochlear’s population mean data were derived from patients’ most recent map in the 3–12-month range. The strong agreement between the 1-month USLs set based on eSRT and the population mean suggests that using eSRT early in one’s programming practice may allow the audiologist to optimize the patient’s map more quickly. This has significant implications for the patient—including shorter time period to improved communication and more rapid trajectory to peak performance—as well as clinical practice optimization and efficiency. As reported by Berg et al (29), eSRT-based programming of USLs at activation or 1-month results in the need for fewer patient follow-up visits as speech recognition reaches asymptote by 3 months. Consequently, this allows for clinical efficiency and more judicious use of healthcare funds by eliminating postoperative visits that are not evidence-based. The data presented herein suggest that the use of population mean in cases for which eSRTs cannot be obtained may yield similar results.

## CONCLUSION

Appropriate setting of USLs is critical to CI recipient outcomes. eSRTs continue to be the recommended, evidence-based method for programming USLs due to the opportunity for individual optimization. When eSRTs are not available, using population mean data provided by Cochlear may serve as a data-driven alternative in close agreement with eSRT-based maps, on average.

## FUNDING SOURCES

This study was supported by NIH UL1 TR000445 and Cochlear Investigator Initiated Research Grant (GR015047).

## CONFLICT OF INTEREST STATEMENT

None directly related to this study. J.H.: consultant for Cochlear and Sonorous; advisory board for Advanced Bionics and MED-EL. R.H.G.: consultant for Advanced Bionics, Cochlear, Skylark Bio, and Sony; audiology advisory board for Advanced Bionics and Cochlear; Institute for Cochlear Implant Training (ICIT) co-CEO clinical board of directors.

## ETHICAL STATEMENT

This study was approved by the institutional review board (IRB# 180939).
